# Reply

**DOI:** 10.4103/0973-6247.42699

**Published:** 2008-07

**Authors:** K. VASANTHA

**Affiliations:** National Institute of Immunohaematology, 13^th^ Floor, NMS Building, KEM Hospital Campus, Parel, Mumbai - 400 012, India. E-mail: dir@icmriihren.nic.in

Dear Sir,

I am herewith giving the answers for the questions put forward by Dr. Manoj Kahar.

1) Monoclonal anti-D reagents are available in the commercial markets as IgM, IgM +IgG and IgG. IgM reagents are used for immediate spin testing and if the immediate spin test is negative, an indirect antiglobulin test has to be performed for confirmation of weak - D using an IgG reagent. An IgM + IgG reagent enables the same test to be carried forward for antiglobulin testing if the immediate spin test is negative.

As majority of our population is Rh D positive, IgM anti-D reagents can be conveniently used for determination of Rh D blood grouping and when it is positive an antiglobulin test is not required in these cases. So only a small percentage of patients and donors need to be confirmed by antiglobulin test. Hence from the available commercial anti-D reagents, the blood banks can decide and buy the required reagents, which fulfill the specifications.

2) (a) The total protein concentration of the anti-D serum can be measured using spectrophotometer and a suitable dilution can be done to make it to 0.2 mg/ml.

(b) and (c) For the preparation of complement coated red cells, you may go through the following reference which gives the methods in detail.

